# Digital economy and the domestic supply chain network

**DOI:** 10.1007/s44265-023-00002-5

**Published:** 2023-04-18

**Authors:** Guangzheng Jing, Shanshan Meng, Miaojie Yu

**Affiliations:** 1 The National School of Development, Peking University, Haidian District, 5 Summer Palace Road, Beijing, 2023 China; 2 The School of Economics, Peking University, Haidian District, 5 Summer Palace Road, Beijing, 2023 China; 3 Liaoning University, Huanggu District, 66 Chongshan Middle Road, Shenyang, 2023 China

**Keywords:** Digital economy, Information transfer, Technological innovation, Supply chain network, D83, F02, O31

## Abstract

Accelerating the development of the digital economy and building digital industrial clusters with international competitiveness has become an important connotation of China's high-quality economic development. From the perspective of the digital economy, this paper makes an in-depth study of the internal mechanism of the development of the digital economy on the supply chain network of Chinese enterprises, and uses the data of Chinese listed enterprises from 2007 to 2016 to identify and test the causal effect and influence mechanism of the digital economy and the position of enterprise supply chain network under the empirical framework. Research shows that the development of the digital economy is conducive to enhancing the influence of Chinese enterprises and improving their central position in the existing supply chain network. The digital economy promotes enterprise supply chain network status through the information transmission effect and technological innovation effect. Compared with digital economy industries, state-owned enterprises, and enterprises in coastal areas, the development of the digital economy has a greater positive role in promoting the position of the supply chain network of traditional industries, non-state-owned enterprises, and enterprises in inland areas. The research of this paper provides a practical basis for accelerating the construction of modern industrial chain and supply chain systems and promoting high-quality economic development.

## Introduction

The COVID-19 pandemic has made it more difficult to match global industrial and supply chains, and “stuck chains” and “broken chains” are spreading around the world. Although China has an independent and complete industrial system with obvious advantages in industrial scale and supporting facilities, and strong resilience in its industrial chain and supply chain, risks of instability and insecurity still exist. Therefore, to ensure the continuous chain of industrial chain and supply chain, from a macro point of view, the whole national economy should strengthen links and smooth circulation, forming an effective supply chain network connecting all links. From the micro point of view, enterprises should improve the production efficiency and core competitiveness of each link in the supply chain network through continuous information sharing and technological innovation. In fact, the current competition among enterprises gradually evolves into the competition of enterprise supply chain network (Christopher 1999).

In recent years, enterprises in the supply chain have gradually evolved from the traditional buying and selling relationship to the complex network cooperation relationship, to compete externally as a whole. In this complex network relationship, more and more enterprises buy the same intermediate input from multiple suppliers, and the customer overlap between enterprises in the same industry increases, which provides opportunities for enterprises to spill technical information through the customer trade network, and also brings new opportunities for enterprises to obtain effective information and narrow the distance between them and the world’s cutting-edge technologies. On the one hand, according to the social relationship network theory (Granovetter 1979), the upstream and downstream enterprises of the supply chain with “strong connections” have close direct business contacts. On the other hand, the “structural hole” theory proposed by Burt et al. (1998) holds that enterprises occupying structural holes in social networks can act as a “bridge” and build a platform for communication between companies that are not directly connected.

The 20th report of the Communist Party of China proposed to build a modern industrial system. We will accelerate the development of the digital economy, promote the deep integration of the digital economy and the real economy, and build internationally competitive digital industrial clusters. Thanks to the development of information and communication technology, the digital economy, as a new economic form, has increasingly become an important driving force for global economic and social development. It not only enables traditional industries in an all-round, all-round and whole-chain way, but also continuously gives rise to new industries and models, thus releasing digital technology's amplification, superposition and multiplication effect on economic development. With the rapid development of digital technology, digital products have become increasingly diverse and become a very important and unique factor input in the production process of enterprises (Branstetter et al. 2019). Goldfarb and Tucker (2019) believe that digital technology reduces the cost in five aspects: search cost, replication cost, transportation cost, tracking cost and verification cost. The digital economy has become an important engine to rebuild the core competitive advantages of manufacturing enterprises, drive real enterprises to improve quality and efficiency, change their driving force, and lead the new growth point of China’s economy. The 14th Five-Year Plan for National Economic and Social Development and the Outline of 2035 Vision Goals of the People’s Republic of China also clearly points out that new advantages of the digital economy should be created to enable the transformation and upgrading of traditional industries. Thus, under the background of the digital economy, how to apply digital economy to the production supply chain network of enterprises has become the economic goal of “innovation-driven development” by using digital technology, and also an important theoretical research issue to achieve “high-quality development” in the 14th Five-Year Plan period of China.

Many scholars have shown that the great development of digital economy supported by digital technology can help enterprises achieve greater efficiency to break through the productivity bottleneck effect, and promote the technological progress of enterprises within the scope of the digital economy (Goldfarb and Tucker 2019), and thus further affect the enterprise supply chain network of enterprises. The digital economy brings the externality of information spillover. Through Internet search, enterprises can break the barrier of “information island”, share research and development results, accelerate the integration of innovative knowledge, improve the scale of R&D investment, and expand the supply chain network of enterprises. It can be seen that the digital economy can enhance the position and influence of enterprises in the supply chain network, enhance the stability of their own supply chain, and reduce the impact and risk of market uncertainties on enterprises by improving their technological innovation ability and information communication ability. However, existing researches mainly analyze the impact of the digital economy on macroeconomic performance, while few researches go deep into Chinese cities to systematically explore the impact of the digital economy on the enterprise supply chain network, which is the core issue of this paper.

In conclusion, compared with the existing literature, this paper tries to make marginal contributions from the following aspects. First, in terms of research topics, this paper examines the impact of the digital economy on the supply chain network of enterprises in China, and explores its internal mechanism in depth. Secondly, in terms of research data, this paper uses the Guotaian Database (CSMAR) from 2007 to 2016 to construct the microcosmic enterprise-level supply chain network index. In addition, databases such as China City Statistical Yearbook and Enterprise Research Data are adopted to construct city-level digital economy index from four dimensions of “digital industry”, “digital innovation”, “digital platform,” and “digital customers”. Finally, in terms of research methods, this paper overcomes the endogeneity problems that may exist in the digital economy by introducing instrumental variables and retesting based on heteroscedasticity identification technology, and the estimated results have good robustness.

## Literature review

With the rapid development of information technology and the advent of the era of the digital economy, the structure of the enterprise-customer trade network tends to be complicated, and the network relationship has gradually become an important channel for enterprises to acquire technical information and realize technological catch-up. In the modern business relationship network system, the core enterprises, upstream suppliers and downstream customers form intricate interrelationships through the division of labor and cooperation of raw materials, spare parts and services, and make the business connection of modern enterprises show significant networking characteristics (Bernard and Moxnes 2018; Di et al. 2018; Bernard et al. 2019). The main form of supply chain network in China is a relational transaction. Under the relationship transaction, enterprises need to make many relationship-specific investments every year to maintain key customers and seek long-term cooperation, which leads to the strong dependence of listed companies on key customers. At present, the emergence and rapid development of digital technology will not only promote the deep integration of various industries, but also may promote the restructuring of the global supply chain. The development of the digital economy extends the horizontal expansibility of enterprise supply chain network through the information communication effect on the one hand, and enhances the vertical competitiveness of enterprise supply chain network through the technological innovation effect on the other hand.

Under the condition of the open economy, information cost is an important part of international trade cost (Rauch 1999; Allen 2014), digital economy provides massive information to exporters at a very low cost, reduces the information cost of exports, and ultimately improves the quality of enterprises’ export products. As a carrier of market information dissemination, the network has attracted the attention of many scholars in the field of trade in terms of helping enterprises overcome transnational information barriers, mainly including the description of the network characteristics of the upstream and downstream of the value chain (Bernard and Moxnes 2018).

The digital economy reduces the cost of information exchange and transaction, thus changing the way of trade and behavior (Anderson and Wincoop 2004; Hellmazik and Schmitz 2015). Anderson and Wincoop (2004) first proposed that communication cost is an important part of trade cost, and the digital economy can reduce communication costs in trade, thus promoting trade development. The explosion of information and data brought by the digital economy and the diversified means of competition have all brought the reduction of operation costs and the improvement of production efficiency to enterprises, which is the network effect given to enterprises (Dunnewijk and Hulten 2007). On the one hand, the information advantages of digital technology help break the business barriers between departments, dredge the barriers of information transmission and enhance timeliness, to improve the operation efficiency of enterprises and reduce management costs. On the other hand, the efficiency of the whole supply chain is crucial to the effective use of enterprise assets and cost reduction. Thanks to the application of digital technology, manufacturing enterprises can quickly obtain and update market information, then quickly provide feedback to the production behavior of manufacturing enterprises, alleviate the production lag caused by information asymmetry and improve production efficiency.

Therefore, digital technology not only promotes the improvement of efficiency through the expansion of production scale on the supply side of manufacturing enterprises, but also expands the economic scope of manufacturing enterprises through the timely transmission of information on the demand side. Therefore, adopting digital technology will bring about a continuous increase in income. High-quality corporate information disclosure can convey information related to corporate value to the outside world, reduce the information asymmetry with external enterprises, alleviate the risk of the stock price crash, reduce the synchronization of stock prices, expand financing channels and improve financing structure (Dan et al. 2016). An important feature of the digital economy is that it compresses the space–time distance and enhances the breadth and depth of inter-regional economic activities through efficient information transmission. Yilmaz et al. (2010) empirically tested panel data of 48 states in the United States, and paid early attention to the spatial spillover effect of informatization. The closer the trade network of the enterprise itself and its neighboring enterprises is connected with the products or markets to be exported, the more beneficial the realization of the potential export relationship of the enterprise. It reflects that the enterprise learns the information of the target trade relationship from its own experience or the relevant experience of other enterprises, which will reduce the uncertainty of the target export market or products and is conducive to the realization of the target trade relationship. Information acquisition has always been one of the important factors affecting enterprise technological innovation and catch-up (Bernard et al. 2019); new information can bring new ideas and technologies to enterprises, and then bring the possibility of higher technical level to enterprises (Keller 2002; Bloom et al. 2013).

Existing studies have shown that digital economy development, such as information technology, Internet development and data management can significantly improve enterprise productivity by reducing communication costs and optimizing resource allocation to improve enterprise production efficiency (Zhong 2018). Some microcosmic enterprise-level studies also found that the digital economy can significantly improve enterprise performance, innovation output and corporate governance efficiency (Prajogo and Olhager 2012; Paunov and Rollo 2016). The emergence of the digital economy accelerates the diffusion and absorption of digital technology, which makes digital technology extend to both ends of the industrial chain and value chain (Guan and Ma 2003), including the vertical derivative industrial chain and the horizontal extension of the value chain, thus possibly improving the innovation capability of enterprises. On the one hand, under the platform effect, the digital economy not only weakens the traditional market boundary and breaks the inter-regional market barriers, but also helps to optimize the innovation environment and improve the matching degree between the liquidity and supply and demand of innovation factors in the industrial chain, which may promote the realization of R & D cooperation between enterprises and enhance the innovation ability of enterprises. On the other hand, the digital economy improves the efficiency of market integration and intensifies competition among enterprises. To regain market competitive advantages, enterprises take the initiative to innovate the way of production organization and the innovation of business model. Basu and Fernald (2010) pointed out that the technology spillover effect first occurred in the information technology production sector and then spread from the information technology production sector to the information technology use sector.

In recent years, the continuous breakthrough of modern information technology such as the Internet and artificial intelligence has created a good environment for establishing the supply-sales relationship between enterprises. The connections between enterprises are increasingly intensive, which not only forms the intricate trade network relationship (Bernard et al. 2019), but also creates more possible channels for inter-enterprise information technology spillover. Through the technology spillover effect, the digital economy realizes and speeds up the popularization and circulation of technology and knowledge among different enterprises, improves manufacturing enterprises' technological level and innovation efficiency, and promotes the improvement of enterprise productivity and enterprise risk-taking. By virtue of information and communication technology, the digital economy can expand the process innovation of various production links of enterprises, promote the formation of positive innovation consciousness of enterprises (Cui et al. 2015), further promote the benign allocation and efficient integration of innovation resources within enterprises, and improve the resource occupancy rate of internal inefficient production links. It can bring about the effect of R & D investment generated by resource reallocation and improve the enthusiasm of enterprises to carry out innovative activities (Czernich et al. 2011). In terms of enterprise innovation behavior, existing studies focus on the influence of interaction between enterprises on the supply chain on knowledge spillover and information sharing behavior, such as knowledge spillover (Isaksson et al. 2016), relationship interaction and network trust (Choi et al. 2020). Isaksson et al. (2016) pointed out that customer innovation has a significant positive impact on supplier innovation, and the degree of customer–supplier relationship embedding plays a positive moderating role. Chu et al. (2019) pointed out that the close geographical distance between suppliers and customers has a significant positive impact on supplier innovation.

## Index construction and research design

### Data

The empirical analysis part of this paper mainly takes listed companies as the research object, with a period from 2007 to 2016. The microcosmic enterprise data used are mainly from the Guo Tai An Database (CSMAR), which contains the basic information, financial information, patent information and other detailed indicators of all listed companies. Other data are mainly from CEPII-BACI Database, China Statistical Yearbook, China City Statistical Yearbook, China E-commerce Yearbook and Enterprise Research Data. On this basis, according to the conventional processing of the data of listed companies in the existing literature, this paper carried out further screening processing on the combined data: (1) Eliminate the sample enterprises that were specially treated by ST or *ST during the sample observation period; (2) Excluding the sample enterprises established in the current year, that is, the operating years of 0; (3) Sample observations excluding the missing or abnormal values of important financial indicators; (4) Sample enterprises that were PT or delisted during the sample period were excluded.

### Indicators and variables

#### Enterprise supply chain network

This paper draws on the social network analysis method to describe and measure the supply chain network at the enterprise level. At the same time, to specifically observe the supply chain network of enterprises in the domestic and international markets, we will construct the indicators of the enterprise’s domestic supply chain network. In social network analysis, researchers often use the network centrality index to measure the central position, influence and closeness of the target object in the network. Most previous studies used the most basic point centrality as the network index, but this index is too simple and may not reflect the specific characteristics of the network. Therefore, Page Rank centrality has been introduced into the research field of economics in recent years. The relative advantage of using this index to measure an enterprise’s supply chain network is that its algorithm is superior to the traditional network measurements indicators such as point degree centrality, intermediate centrality, proximity centrality and feature vector centrality in comprehensively reflecting supply chain network correlation and weakening the influence of other node centrality.

PageRank centrality comes from Google’s webpage ranking index, which mainly reflects the importance of a webpage affected by the number and quality of links to other pages. Specifically, Google’s ranking of sites takes into account that users are directed between web pages by hyperlinks. A jump from Web page A to Web page B is not directed from Web page B to Web page A. The jump from a popular web page should be more important than a jump from a less popular web page. This is the essence of the PageRank algorithm. It can also be found that the idea basis of the algorithm is consistent with the centrality of feature vectors. In addition, PageRank centrality can not only measure the relative importance of a webpage, but also be used to measure search, traffic and other indicators (Page et al. 1999).

The formula for calculating PageRank centrality is:

1 $${PageRank}_{i}=\alpha \sum_{j-1}^{N}{A}_{j,i}\frac{PageRank}{{d}_{out,j}}+\frac{1-\alpha }{N}$$

where, $PageRank$ represents the value of PageRank centrality of the enterprise. $N$ represents the total number of enterprises (nodes) in the entire supply chain network. ${A}_{j,i}$ is the adjacency matrix whose order is $N\times N$, it represents the state of business between enterprises in the supply chain network. ${d}_{out,j}$ represents the total number of cooperative objects of the enterprise $j$. $\alpha$ stands for damping coefficient. Consistent with the empirical value of the PageRank algorithm, 0.85 was taken as the damping coefficient value of this algorithm (Brin and Page 1998).

It can be seen from the calculation formula of PageRank centrality that the calculation of this index needs to be iterated several times through the correction rule and finally converges to a stable value. The specific iterative process will not be described in this article. In addition, the total sum of the PageRank centrality of each enterprise in the network is 1, ${\sum }_{i}{PRC}_{i}=1$.

Domestic supply chain network($PageRank\_d$). This paper uses two data sets of the top five customers and the top five suppliers of listed companies to construct the domestic oriented weighted supply chain network of listed companies to depict the domestic supply chain network relationship of listed companies. In this database, some listed companies did not publish the specific enterprise names of their top five customers or suppliers, and used similar words such as “customer (supplier)” or “the first largest customer (supplier)” instead. Considering that the data quality may affect the accuracy of the research conclusions, this paper eliminated the data of such enterprises that could not be accurately identified. Based on this database, formula (1) is used to construct and calculate the index of domestic supply chain network($PageRank\_d$).

#### Digital economy

The digital economy first appeared officially in the 1990s and was soon popular the academic circles. However, different literatures refer to different digital economies, even though most are related to Internet technology. With the rapid development of new digital technologies such as the Internet of Things, big data, artificial intelligence and blockchain, The simple “Internet economy” has been difficult to continue to become the synonym of the digital economy, but so far in the academic circle there is still no unified definition and measurement standards. At present, the definition given in the G20 Initiative on the Development and Cooperation of Digital Economy is widely recognized: digital economy is a series of economic activities in which digital knowledge and information are the key factors of production, modern information network is an important carrier, and effective use of information and communication technology is an important driving force for improving efficiency and optimizing economic structure. Bukht and Heeks (2018) divided the definition of the digital economy into three levels. The core layer is about the level of digital infrastructure, the middle layer includes digital services and platform economy, and the outermost layer includes e-commerce, algorithm-driven economic activity, etc. According to this definition and in view of data availability, this paper measures the digital economy from the four dimensions of digital industry activity, digital innovation activity, digital platform activity and digital user activity. The KMO test value of the four dimensions of digital economy indexes sorted out in this paper is greater than 0.85, which meets the necessary conditions of principal component analysis. Therefore, we use the principal component analysis method to reduce the dimensionality of 13 indicators of digital industry activity, digital innovation activity, digital user activity and digital platform activity after standardization, and integrate them into a comprehensive digital economy index $dige\_pca$ (Table 1).

**Table 1:** Digital Economy Sub-index

Dimensionality	Variables	Data Sources	Data Level
Digital Industry	share of employment in information transmission, computer services and software	China Urban Statistical Yearbook	Prefecture- Level City
	Information transmission, computing and service and software industries account for the proportion of fixed assets in the whole society	China Statistical Yearbook	Provincial Level
	Software revenue	China Statistical Yearbook	Provincial Level
Digital Innovation	number of patents granted to the industrial internet industry	Enterprise research data—Industrial Internet special database	Prefecture- Level City
	number of patents granted in e-commerce industry	Enterprise research data—special database of e-commerce industry	Prefecture- Level City
	number of patents granted in the 5G industry	Enterprise research data-5G industry special database	Prefecture- Level City
Digital Platform	number of Internet users	Statistical report on the development of China's Internet	Provincial Level
	number of websites		Provincial Level
	number of domain names		Provincial Level
Digital Subscriber	mobile phone penetration	China Urban Statistical Yearbook	Prefecture- Level City
	total volume of telecommunication service		Prefecture- Level City
	per capita number of Internet broadband users		Prefecture- Level City
	e-commerce transaction volume	Statistical Yearbook of China Electronic Information Industry	Provincial Level

Given the data availability, we construct the above digital economy index at the city level, which is also the most widely used and specific level at present. Considering that our explained variable is the supply chain network at the enterprise level, this paper uses text analysis to analyze the digital transformation index of enterprises. The first step is to collect and convert the annual reports of listed manufacturing companies from 2007 to 2016 into text format, and then extract the text of the analysis part of business situation through Python. The second step is to conduct word segmentation and word frequency statistics on the selected samples, screen out the high-frequency words related to digital transformation and make word cloud map, which can be divided into four dimensions: digital technology application, Internet business model, intelligent manufacturing and modern information system. This suggests that we can construct the digital transformation index of enterprises from four dimensions. Third, based on the words formed in the second step, extract the text before and after the total sample of listed companies, and find the text combination with high frequency. Fourth, based on the self-built word segmentation dictionary, Jieba function is used to segment all samples and count the number of keyword disclosure from the four aspects of digital technology application, Internet business model, intelligent manufacturing and modern information system, so as to reflect the transformation degree of enterprises in all aspects. The fifth step is to standardize the word frequency data, use the entropy method to determine the weight of each index, and finally get the DIGI_text index. Although relevant studies have been carried out using this index, the interpretation of the definition of digital economy of this index is not as rich as that of the index at the prefecture level, so this paper finally chooses the digital economy index at the prefecture level as the core explanatory variable of the benchmark regression. Furthermore, the robustness test of the digital economy index at the enterprise level is carried out to ensure the robustness of the benchmark regression and the credibility of the research conclusions of this paper.

#### Control variable

To control the endogenous problems caused by missing variables that may affect the enterprise supply chain network, control variables at the enterprise level are added in this paper, which mainly include the operating years of the enterprise (lnage), the size of the enterprise (lnscale), the return on assets (roa) and the operating income of the enterprise (lnrev). In addition, to control the bias of the estimation results brought by the differences between firms that do not depend on time variables, we also control the fixed effects at the firm (${\gamma }_{i}$) and time (${\gamma }_{t}$) levels, and use the firm level clustering standard error.

See Table 2 for the meaning and descriptive statistics of each variable and the specific situation of sub-indexes of the digital economy.

**Table 2:** Descriptive Statistics of Variables

Variable Symbol	Variable Meaning	Observed Value	Mean Value	Standard Deviation
$PageRank\_d$	The centrality of enterprise domestic supply chain network	9994	0.19	0.24
$dige\_pca$	digital economy index	13,594	0.93	1.08
ln*age*	operating life of enterprise	13,374	2.65	0.42
ln*scale*	enterprise scale	14,440	7.58	1.30
*roa*	return on assets	14,091	0.04	0.24
ln*rev*	business income	14,083	21.20	1.49

## Empirical results and analysis

### Basic regression

Table 2 reports the results of baseline regression in this paper. Among them, the core explanatory variables are added to column (1), including fixed effects of industry and year. On this basis, the fixed effects at the level of enterprise and time are further added to column (2), and other control variables continue to be added to column (3). It is worth noting that when industry and, year and firm fixed effects and other control variables are added respectively, the influence of digital economy on enterprises' domestic supply chain network is always significantly positive. This paper will take the estimation results in column (3) as the representative for analysis. In summary, the digital economy level indicators are significantly positive at the 1% level. When the digital economy level increases by one unit, the PageRank centrality of the enterprise in the domestic supply chain network increases by 23.32%, that is, the improvement of the digital economy level is conducive to the development of the enterprise supply chain network. This is because the improvement of the level of digital economy means that the Internet, artificial intelligence and other modern information technology is highly developed, which will provide a good environmental basis for the establishment and development of enterprise supply chain network. On the one hand, the development of digital economy greatly reduces the cost of information friction when enterprises search for customers or suppliers, which will accelerate the establishment and expansion of enterprise supply chain network. On the other hand, the development of digital economy accelerates the transfer of heterogeneous technical resources, improves the level of technological innovation of enterprises, and enables enterprises to have greater autonomy in the selection of customers and suppliers. At this time, their customers and suppliers are more inclined to maintain cooperative relations with them, and they are more difficult to be replaced as business partners of other enterprises. That is, the digital economy helps enterprises to enhance their influence and improve their central position in the existing supply chain network (Table 3).

**Table 3:** Results of Baseline Regression

	(1)	(2)	(3)
*dige_pca*	0.2205^a^ (0.0711)	0.2847^a^ (0.0654)	0.2332^a^ (0.0707)
CV_firms	NO	NO	YES
Firm fixed effect	NO	YES	YES
Time-fixed effect	YES	YES	YES
Industry-fixed effect	YES	NO	NO
Cons	-0.8771 (1.0647)	2.8688^a^ (0.0641)	-1.2956^a^ (1.0297)
N	6820	7229	6822
adj.R^2^	0.4588	0.4515	0.4464

*Note*: Cluster robust standard error in brackets; ^a^ denote, respectively, significance at 0.01 levels

### Mechanism analysis

This paper argues that, on the one hand, the development of the digital economy can accelerate the information transmission of the trading market, and reduce the degree of information asymmetry between enterprises, to promote the development of enterprises’ domestic supply chain, that is, the information transmission effect; On the other hand, it can improve the technological innovation level of enterprises, thus affecting the establishment and expansion of enterprise supply chain, namely the technological innovation effect. Based on this, this paper further tests the above two possible influence channels. In the way of the mechanism test, this paper uses mechanism variable as explained variable for regression.

#### Information transfer effect

In this paper, two indicators are used to measure corporate information transparency to observe the information transmission situation of enterprises. One is the amount of tracking by analysts, and the other is the quality rating of information disclosure. The tracking number of analysts comes from the index of listed companies’ attention by analysts in Guo Tai An database—the number of analysts who track and analyze the listed companies within the sample year. When the tracker is an analyst team, the tracking number is regarded as 1; The information disclosure rating comes from the information disclosure assessment results of Shenzhen Stock Exchange and Shanghai Stock Exchange. The assessment content mainly includes the timeliness, accuracy, completeness and legality of information disclosure. The assessment rating is divided into four grades, namely excellent (A), good (B), qualified (C) and unqualified (D). In this paper, the variable is further treated as 0–1 variable, that is, when the assessment rating is A and B, we believe that the enterprise information disclosure quality is higher, that is, the information transparency is higher, which will be more conducive to the information transfer between enterprises. The value of the information transfer variable is 1, When the assessment rating is C or D, the value is 0.

The estimated results are shown in columns (1) and (2) of Table 4. In column (1), the logarithm of analyst tracking quantity is used as the mechanism variable, and in column (2), the quality of information disclosure is used as the mechanism variable. The estimated results show that for information transparency measured by different variables, the coefficients of the digital economy are all significantly positive. It shows that the digital economy's development has significantly improved enterprise information transparency. On the one hand, the development of the digital economy mainly relies on the rapid development of the Internet. On the other hand, “Internet + ” technology provides more network communication platforms for enterprises, diversifies the communication channels between enterprises and potential business partners, and significantly improves communication efficiency and corporate information transparency. On the other hand, in the era of the digital economy, emerging technologies such as big data and 5G commercial use are actively applied in all walks of life. In this context, the information related to enterprises will also be accelerated across the country and the world. To further improve the efficiency of matching with potential business partners, enterprises will also be encouraged to improve their information transparency.

**Table 4:** Results of Mechanism Test

	(1)	(2)	(3)
	lnfxs	disclosure	rd_ratio
dige_pca	0.0764^b^ (0.0220)	0.0268^a^ (0.0147)	0.2330^a^ (0.1057)
CV_firms	YES	YES	YES
Firm fixed effect	YES	YES	YES
Time-fixed effect	YES	YES	YES
cons	-2.9378^b^ (0.5109)	4.6714^b^ (0.3040)	32.5564^b^ (3.4180)
N	9740	12,539	7586
Adj.R^2^	0.5823	0.8642	0.7420

*Note*: Cluster robust standard error in brackets; ^a^, and ^b^ denote, respectively, significance at 0.10, and 0.01 levels

#### Technological innovation effect

This paper uses the R & D investment ratio (the proportion of R & D investment to total assets) of listed companies to measure the innovation level of enterprises. Considering that the digital economy may directly affect the innovation decisions of enterprises when it affects enterprise innovation, that is, how much money enterprises invest in the research and development of new products and technologies, there may be a certain degree of lag in the impact of enterprise innovation output, namely innovation patents, especially for the invention patents with greater innovation difficulty and higher level. There may be different degrees of the time lag from the beginning of research and development to patent application and patent authorization, and appearance patents and design patents with short research and development time are not representative of enterprise innovation level. Therefore, this paper finally chooses to use the proportion of enterprise R&D investment to measure the change of enterprise innovation level.

The test results of the technological innovation effect are reported in Table 4 (3). We find that the estimated coefficient of digital economy variables is significantly positive, indicating that the digital economy has indeed played a significant role in promoting enterprise innovation. Based on the concept of digital economy, the development of digital economy mainly relies on developing and applying emerging technologies. Therefore, it is easy to understand that the digital economy's development can improve enterprises' technological innovation ability, and the deeper reasons can be roughly summarized into three aspects. First, in the era of the digital economy, enterprises can timely receive product and customer information through the application of digital technology. Then accelerate the iterative innovation of products and technologies according to the market feedback results to match the consumer demand of users; Secondly, the development of the digital economy provides convenience for enterprises to obtain the latest information resources and technical resources effectively. Relying on “Internet + ”, enterprises can quickly master new skills to increase the investment in technological innovation and improve their innovation ability. Third, the development of digital economy tends to promote the entry of new enterprises, and the competition among enterprises becomes more fierce, which will force enterprises to increase innovation investment and improve the level of technological innovation, because in this environment, if enterprises do not improve their innovation ability, they cannot survive in the fierce competition in the era of the digital economy in the long term. In addition, the digital economy can accelerate the informatization process of enterprises, realize the digital transformation and upgrading of enterprises through the application of emerging technologies, and enhance the innovation capability of enterprises. In addition, existing studies have also confirmed the promoting effect of the digital economy on the number of enterprise patents, providing additional supplementary evidence for the estimated results of this paper.

### Robustness test

Benchmark regression results show that the development of the digital economy has a significant role in promoting the position of enterprises’ domestic supply chain network. To ensure the credibility of the regression results in this paper, a series of robustness tests will be conducted from the following aspects. First of all, for the possible missing variables and endogeneity problems caused by mutual causal relationship, we introduced instrumental variables and heteroscedasticity based identification technology to conduct a re-test. Secondly, considering that the result of benchmark regression may be affected by the definition of enterprise supply chain network index and digital economy development index, we will change the method to re-measure core explanatory variables and dependent variables.

#### Endogenous problem

The validity of the benchmark regression results in this paper may be faced with missing variables and mutual causality. On the one hand, although fixed effects at the firm level and time level are controlled in this paper to avoid differences between firms that do not change with time, there may still be other invisible missing variables that cause bias in the estimation results. On the other hand, the improvement of the digital economy has strongly promoted the development of the enterprise supply chain network. On the contrary, although the explanatory and explained variables in this paper are city level and enterprise level respectively, the expansion of the enterprise’s domestic supply chain network has enriched the enterprise's information network and will bring the latest information technology resources in the world to the location of the enterprise. Promote the development of local digital economy. Based on this, to overcome the possible endogeneity problem and realize causality inference, the instrumental variable method and heteroscedasticity based recognition technique method will be used respectively for testing.

In terms of the selection of instrumental variables, this paper uses historical post and telecommunication data and geographical location data as exogenous Shift-Share instrumental variables (Goldsmith-Pinkham et al., 2020). First of all, this paper uses the total volume of post and telecommunications business of each prefecture-level city in 1985 as the instrumental variable of the digital economy to measure the development basis of the digital economy of each city in history. On the one hand, post and telecommunications services can generally be regarded as the reflection of a region’s demand for information communication, and the historical demand for information at the regional level will generally affect the development of information technology in the subsequent stage. Meanwhile, the index of total telecommunications services is also included in the construction of digital economy development indicators in this paper, so this variable meets the conditions of relevance. On the other hand, the index year we selected is 1985, which is relatively far from the sample period used in this paper, and postal and telecommunication services in history will not directly affect the development of the supply chain network of enterprises in the later period, so this index meets the strict exogenous conditions. It is worth noting that since the historical data of posts and telecommunications are cross-sectional data that do not change with time, Therefore, this paper takes the logarithm of the number of Internet users in China as the shift part of shift-share and multiplicative (ln(y_1985)*ln(num_w)) of the historical data of posts and telecommunications as the benchmark instrumental variable changing over time.

To further ensure the robustness of the test results of the above instrumental variables, this paper continues to use the interaction term (ln(dist)*ln(num_f)) of the logarithm of the distance between the enterprise and the coastal port (share) and the logarithm of the stock of the national digital economy enterprise (shift) as the second instrumental variable for retest. Theoretically speaking, the closer an enterprise is to a coastal port, the higher the level of economic development where the enterprise is located, which is more conducive to the development of the digital economy. While the total stock of digital economy enterprises nationwide reflects the development trend of the digital economy nationwide. Undoubtedly, the interaction term between the two is highly correlated with the development level of the regional digital economy. The two are not directly related to enterprises' current supply chain network development, because the instrumental variables also meet the conditions of “strong correlation” and “strict externality”.

Table 5 reports the estimation results of the two instrumental variables. Among them, the LM statistics of Kleibergen-Paap rk reject the null hypothesis at the 1% level, that is, both of the two instrumental variables meet the discernibility. Meanwhile, the Wald F statistics of Kleibergen-Paap rk are far greater than the critical value of the Stock-Yogo weak identification test, that is, there is no problem of weak instrumental variables. The above test indicates that the two instrumental variables selected in this paper are reasonable and feasible. Further, according to the estimation results of the second stage of the test of two instrumental variables, the coefficients of the digital economy variables are significantly positive, indicating that the digital economy has significantly promoted the domestic development of enterprises, further confirming the conclusion of the benchmark regression results.

**Table 5:** Endogenous Problem—Instrumental Variables

	(1)	(2)
	First Stage *dige_pca*	Second Stage Pagerank_d
*dige_pca*		3.1395^a^ (0.7108)
ln(*num_w*)*ln(*y_1985*)	0.6568^a^ (0.0734)	
CV_firms	YES	YES
Firm fixed effect	YES	YES
Time-fixed effect	YES	YES
Kleibergen-Paap rk LM statistic		60.86 (0.0000)
(Kleibergen-Paap rk Wald F statistic		80.16 (16.38)
N	6519	6519
R^2^		1.2440
	(3)	(4)
	First Stage *dige_pca*	Second Stage Pagerank_d
*dige_pca*		2.5528^a^ (0.5344)
ln(*dist*) *ln(*num_f*)	0.0229^a^ (0.0017)	
CV_firms	YES	YES
Firm fixed effect	YES	YES
Time-fixed effect	YES	YES
Kleibergen-Paap rk LM statistic		84.98 (0.0000)
(Kleibergen-Paap rk Wald F statistic		106.92 (16.38)
N	6815	6815
R^2^		1.1560

*Note*: Cluster robust standard error in brackets; ^a^ denote, respectively, significance at 0.01 levels

In addition to the instrumental variable method, this paper also refers to the recognition technology based on heteroscedasticity proposed by Lewbel (2012) to solve the potential endogeneity problem, generally used in the failure to find appropriate instrumental variables, but also as a further supplement to the instrumental variable method. This method breaks through the conditions related to exclusion restriction of the instrumental variable method and only needs to meet the conditions that the errors are heteroscedasticity during use. The test results are shown in Table 6. We find that the positive impact of the digital economy on the domestic supply chain network of enterprises is still significant and robust, which is consistent with the benchmark regression results and the estimation results of the above two instrumental variables, indicating the reliability of the research conclusions in this paper.

**Table 6:** Endogenous Problem—Identification Technique Test Based on Heteroscedasticity

	(1)
dige_pca	1.7368^a^ (0.7472)
CV_firms	YES
Time-fixed effect	YES
N	7166
Adj.R^2^	0.4216

*Note*: Cluster robust standard error in brackets; ^a^ denote, respectively, significance at 0.05 levels

#### Replacement core metrics

To further test the robustness of the estimation results, the characteristic indicators of enterprise supply chain network and digital economy indicators are replaced in this paper, and the estimation results are shown in Table 7. This paper re-examined the degree of centrality (degree_d) most commonly used in network analysis in domestic supply chain networks. The estimates in column (1) of Table 7 show that the digital economy significantly promoted the development of domestic supply chain networks. In terms of digital economy indicators, we used the word frequency number related to digital economy extracted by text analysis method to construct the enterprise-level digital economy indicator (dige_wenben) for re-testing. The estimated results in column (2) of Table 7 show that the digital economy can significantly promote the development of the domestic supply chain network of enterprises, which is consistent with the benchmark regression results.

**Table 7:** Change core variable index

	(1)	(2)
	replace supply chain network indicators	replace digital economy indicators
dige_pca	0.4877^a^ (0.1298)	
dige_wenben		1.9696^a^ (0.4256)
CV_firms	YES	YES
Firm fixed effect	YES	YES
Time-fixed effect	YES	YES
cons	0.6290 (2.0793)	-1.4927^a^ (1.0156)
N	6822	7046
Adj.R^2^	0.4869	0.4411

*Note*: Cluster robust standard error in brackets; ^a^ denote, respectively, significance at 0.01 levels

### Heterogeneity analysis

Considering that heterogeneous characteristics of enterprises (such as industry characteristics, ownership characteristics and local characteristics) may affect the promoting effect of the digital economy on enterprise supply chain network, we conducted heterogeneity analysis based on different industries, ownership and regions of enterprises to investigate the impact of the digital economy on enterprise supply chain network more comprehensively and completely.

#### Industry heterogeneity (Table 8)

**Table 8:** Regression Results of Industry Heterogeneity

	(1)	(2)
	digital economy industry	traditional industry
dige_pca	-0.1907 (0.1160)	0.1545^a^ (0.0872)
CV_firms	YES	YES
Firm fixed effect	YES	YES
Time-fixed effect	YES	YES
cons	6.2655^b^ (2.6989)	3.0820^b^ (1.4450)
N	1292	5530 0.4576
Adj.R^2^	0.4638	

*Note*: Cluster robust standard error in brackets; ^a^, and ^b^ denote, respectively, significance at 0.10 and 0.05 levels

#### Ownership heterogeneity

This paper divides the samples into state-owned enterprises and non-state-owned enterprises according to the ownership attributes of enterprises for heterogeneity analysis. Due to its special attributes, state-owned enterprises generally have larger enterprise scale, lower financing constraints and stronger political relevance, so they have more ways and more efficient access to the latest information and cutting-edge technology resources, and are more capable of expanding their supply chain network. In contrast, non-state-owned enterprises, especially private enterprises, are faced with more constraints and challenges. When searching for new customers and suppliers, they often face serious information asymmetry, which will lead to high information search costs and is not conducive to the establishment and expansion of the enterprise supply chain network. The specific estimation results are shown in Table 9. First, in the sample of non-state-owned enterprises, the estimation coefficient of the digital economy index is significantly positive, indicating that the digital economy does promote the development of the domestic supply chain network of non-state-owned enterprises, which is consistent with the expectation of this paper. Secondly, the regression results of the samples of state-owned enterprises show that the influence of the digital economy on the domestic supply chain of state-owned enterprises is insignificant. The possible reason is that this paper believes that the influence of the digital economy on the supply chain network of enterprises is mainly the effect of information transmission, while state-owned enterprises have a strong ability to obtain information in China, so they are not strongly dependent on the development of the digital economy.

**Table 9:** Regression Results of Ownership Heterogeneity

	(1)	(2)
	state-owned enterprise	non state-owned enterprises
dige_pca	0.1796 (0.1273)	0.1703^b^ (0.0776)
CV_firms	YES	YES
Firm fixed effect	YES	YES
Time-fixed effect	YES	YES
cons	5.7465^b^ (2.4923)	2.0172^b^ (0.7092)
N	3232 0.4788	5530 0.0882
Adj.R^2^		

*Note*: Cluster robust standard error in brackets; ^a ^and ^b^ denote, respectively, significance at 0.10 and 0.05 levels

#### Regional heterogeneity

According to the region of the enterprise (prefecture-level city), this paper divides the two sub-samples into coastal areas and inland areas for regression test, and the results are shown in Table 10. It can be seen that the digital economy has a greater positive impact on the supply chain network of enterprises in inland areas. The reason is that compared with inland areas, the eastern region has a better geographical location, more complete infrastructure, more convenient transportation, higher degree of openness, and more abundant factor resource endowment. These natural or historical advantages facilitate the development of the supply chain network of enterprises in coastal areas, and they are less dependent on the development of the digital economy. Enterprises in inland areas face a worse overall market environment. The development of the digital economy reduces the cost of information search and improves the innovation ability of enterprises, which undoubtedly provides new development opportunities and transformation and upgrading power for enterprises. Therefore, the digital economy plays a crucial role in the development process of the supply chain network of enterprises in inland areas.

**Table 10:** Results of regional heterogeneity regression

	(1)	(2)
	coastal region	inland region
dige_pca	0.0960 (0.1101)	0.2997^a^ (0.0987)
CV_firms	YES	YES
Firm fixed effect	YES	YES
Time-fixed effect	YES	YES
cons	-2.3642 (1.7095)	-0.6510 (1.2499)
N	2215	4607
Adj.R^2^	0.4238	0.4566

*Note*: Cluster robust standard error in brackets; ^a ^denote, respectively, significance at 0.01 levels

## Conclusion and enlightenment

By constructing the latest digital economy index, this paper systematically combes the digital economy’s mechanism on the supply chain network of microcosmic enterprises in China. This paper uses the data of Chinese listed enterprises from 2007 to 2016 to carry out the corresponding empirical test. In summary, this paper mainly draws the following conclusions: First, the better the development of the digital economy, the more favorable it will be for Chinese enterprises to enhance their influence and improve their central position in the existing supply chain network; Secondly, according to the mechanism test, digital economy promotes the improvement of enterprise supply chain network status through information transmission effect and technological innovation effect. Thirdly, heterogeneity analysis shows that, compared with digital economy industries, state-owned enterprises and enterprises in coastal areas, the development of the digital economy has a greater positive promoting effect on the supply chain network status of traditional industries, non-state-owned enterprises and enterprises in inland areas.

From the perspective of the digital economy, this paper makes an in-depth study of the impact of the development of the digital economy on the position of Chinese microcosmic enterprises in the supply chain network, and obtains the following policy implications: First, it is necessary to establish a smooth, effective, flexible and orderly national supply chain market, give full play to the positive role of the digital economy in optimizing the market-oriented allocation efficiency of factors, and effectively improve the position of enterprises in the supply chain network. Secondly, new digital economy technologies such as artificial intelligence, big data and blockchain should be used to improve enterprises’ information communication ability and technological innovation ability, and constantly improve the scalability and stability of their domestic supply chain network. Finally, it is necessary to meet the digital transformation needs of enterprises in traditional industries, non-state-owned enterprises and enterprises in inland areas in a targeted way, strengthen the digital empowerment and innovation transformation of various enterprises, to further enhance the innovation vitality of the industrial chain and supply chain and the efficiency of resource allocation, and accelerate high-quality economic development.

## Data Availability

All data generated or analysed during this study are included in this published article in the reference section.
